# 25-Hydroxyvitamin D status, metabolic syndrome and insulin resistance in preadolescent child in Korea

**DOI:** 10.1186/1687-9856-2013-S1-P96

**Published:** 2013-10-03

**Authors:** Hae Soon Kim, Hye Ah Lee, Young Ju Kim, Eun Ae Park, Hyesook Park

**Affiliations:** 1 Department of Pediatrics, School of Medicine, Ewha Womans University, Seoul, Korea; 2 Department of preventive medicine, School of Medicine, Ewha Womans University, Seoul, Korea; 3 Obstetrics and Gynecology, School of Medicine, Ewha Womans University, Seoul, Korea

## Background and aims

The lower 25-Hydroxyvitamin D(25(OH)D) is being suggested as a risk factor for Type 2 diabetes, but there is not well construed for its relationship. Therefore, we aimed to assess the association among 25(OH)D level, metabolic syndrome components [Waist circumference(WC), Blood pressure, Triglycerides, Glucose, High-Density lipoprotein Cholesterol], and insulin resistance indices [homeostasis model assessment-insulin resistance, quantitative insulin-sensitivity resistance, glucose to insulin ratio] among preadolescent child.

## Methods

We followed up 221 subjects from July to August 2011 aged 7 to 9 child, who were part of Ewha Birth & Growth Cohort study, Seoul, Korea, which is a prospective cohort established 2001-2006. We investigated the associations among vitamin D level in blood, metabolic syndrome components, and insulin resistance using multivariate regression analysis adjusted for sex, age, birth weight, calories, and BMI z score.

## Results

38(17.2%) child were deficiency (<20ng/mL) for vitamin D level, and its prevalence was more higher in boys (25.2%) than girls (8.1%). There was significant relationship between 25(OH)D and triglycerides(*β*=-0.01, *p*=0.04) adjusted for sex, age, birth weight, calories, and BMI z score. WC was also negatively associated with 25(OH)D (*β*=-0.13, *p*=0.07) with cofactors, although its relationship was shown the marginal boundary. But, other features were not associated with 25(OH)D. When regarding the criteria for metabolic components, those who were more than WC 90%tile had higher frequency in deficient group than in sufficient group, but distribution of metabolic components among 25(OH)D status was no significant difference.

## Conclusions

The lower 25(OH)D level may contribute to the association with some of metabolic components in general preadolescent child, but further study is needed to explore the relationship with insulin resistance.

